# Uncovering the semantics of concepts using GPT-4

**DOI:** 10.1073/pnas.2309350120

**Published:** 2023-11-30

**Authors:** Gaël Le Mens, Balázs Kovács, Michael T. Hannan, Guillem Pros

**Affiliations:** ^a^Department of Economics and Business, Universitat Pompeu Fabra (UPF), Barcelona School of Economics (BSE), UPF-Barcelona School of Management, Barcelona 08005, Spain; ^b^Yale School of Management, Yale University, New Haven, CT 06520; ^c^Stanford Graduate School of Business, Stanford University, Stanford, CA 94305

**Keywords:** categories, chatGPT, deep learning, typicality, LLM

## Abstract

The ability of recent Large Language Models (LLMs) such as GPT-3.5 and GPT-4 to generate human-like texts suggests that social scientists could use these LLMs to construct measures of semantic similarity that match human judgment. In this article, we provide an empirical test of this intuition. We use GPT-4 to construct a measure of typicality—the similarity of a text document to a concept. We evaluate its performance against other model-based typicality measures in terms of the correlation with human typicality ratings. We conduct this comparative analysis in two domains: the typicality of books in literary genres (using an existing dataset of book descriptions) and the typicality of tweets authored by US Congress members in the Democratic and Republican parties (using a novel dataset). The typicality measure produced with GPT-4 meets or exceeds the performance of the previous state-of-the art typicality measure we introduced in a recent paper [G. Le Mens, B. Kovács, M. T. Hannan, G. Pros Rius, *Sociol. Sci.* **2023**, 82–117 (2023)]. It accomplishes this without any training with the research data (it is zero-shot learning). This is a breakthrough because the previous state-of-the-art measure required fine-tuning an LLM on hundreds of thousands of text documents to achieve its performance.

## Significance

We use GPT-4 to create “typicality measures” that quantitatively assess how closely text documents align with a specific concept or category. Unlike previous methods that required extensive training on large text datasets, the GPT-4-based measures achieve state-of-the-art correlation with human judgments without such training. Because training data is not needed, this dramatically reduces the data requirements for obtaining high performing model-based typicality measures. Our analysis spans two domains: judging the typicality of books in literary genres and the typicality of tweets in the Democratic and Republican parties. Our results demonstrate that modern Large Language Models (LLMs) can be used for text analysis in the social sciences beyond simple classification or labelling.

Are politicians who express opinions that differ from the prevalent views of their political party penalized for doing so? Do consumers prefer cultural items that fall into an established genre or those that challenge established norms (1)? When companies seek to fill a position, do they hire job candidates with a career identity that is typical of the position or those with more atypical experiences (2, 3)? Do research articles which conform to the standards of specific fields have a larger impact on subsequent research (4)? Are patent applications that propose a technology that clearly fits within a technology class more likely to be approved by patent examiners?

Answering these questions requires, in part, determining the semantic similarity between objects and concepts (or categories). Research in social and behavioral science refers to such an assessment as “perceived typicality.” Typicality in a concept is the degree to which an object is perceived as representative or prototypical of that concept (5). For instance, a typical Mystery book might be thought of as a suspenseful and engaging piece of fiction that challenges readers to solve the puzzle with the protagonist. A book with these characteristics will likely be judged to have a high typicality in the Mystery concept, while one lacking these features will be judged to have a lower typicality.

In many settings of interest to social and behavioral scientists, objects are often described through text. In such settings, typicality is a measure of semantic similarity between a text document and a concept.

In this article, we propose to construct typicality measures from text data by directly asking GPT-4 for a typicality rating through a chat completion request via the API. For example, if we are interested in measuring the typicality of a book in the Mystery literary genre based on its description like those commonly found on back covers or on review websites such as Goodreads.com or Amazon.com, we use the following prompt:

Here’s a book description: “Ten-year-old Jack McGurk and his fellow detectives match wits with the local police as they track down a runaway newsboy.” How typical is this book of the “Mystery” genre? Provide your response as a score between 0 and 100 where 0 means “Not typical at all” and 100 means “Extremely typical.”

We submit this query to GPT-4 several times via the OpenAI API and take, as the typicality measure, the arithmetic average of the numerical scores returned by the language model. This typicality measure improves on the state-of-the-art measure we recently introduced (6) in terms of its correlation with the typicality ratings of human judges. Crucially, it achieves this performance with zero-shot learning: The model was not trained to make typicality predictions in the focal concept (the Mystery literary genre in the above example). Our approach relies on the text generation capabilities of pretrained, off-the-shelf GPT-4.

In contrast, the previous state-of-the-art required fine-tuning BERT a LLM introduced in 2018 (7) on a large amount of research data (several hundred thousand book descriptions together with genre labels, in the case of typicality in the Mystery genre (6)). Therefore, the typicality measure produced with GPT-4 is both more economical in terms of data requirements and more empirically valid than the prevailing methods. Our approach is potentially applicable to research settings in which there are not enough data available to assemble a training set.

Next, we briefly review the related literature and analyze the performance of the typicality measure produced with GPT-4 in terms of its correlation with human typicality ratings on the test data used in our previous article (6) and a dataset of tweets published by members of the US Congress collected for this article. The body of the article focuses on the comparison with the current state-of-the-art model-based typicality measure based on BERT. Comparisons with measures based on GPT-3, GPT-3.5 Turbo, and GPT-3 text embeddings are reported in *SI Appendix*.

## Related Literature

Issues of measuring typicality arise routinely in studies of social and economic organization. In the study of organizations and markets, the focus has been on how agents acting as audience members judge the offers of producers. A crucial part of the evaluation process involves evaluating the fit of producers and their products with the prevailing categories (8–12). In other words, concepts such as industry, product category, and cultural genre form the basis for audience expectations, and the degree to which producers and their products are typical of established categories indicates whether they deserve attention. A similar process affects how employers evaluate potential job candidates (1).

It is important to note that measuring the typicality of an object in a concept is not the same task as categorizing an object. The main reason is that, even though typicality and category membership are related constructs, they are distinct. Typicality refers to the internal structure of concepts (5, 12, 13). As an illustration, note that both an apple and an avocado are fruits, but people generally perceive an apple as a more typical fruit than an avocado (5). Producing a typicality measure involves producing a numerical estimate that characterizes the fit of an object to a reference concept rather than choosing between a set of candidate concepts.

Early sociological studies of typicality generally analyzed only the categorizations of objects, not their feature values (1, 8, 9, 14, 15). For example, (16) studied restaurant cuisines and assumed that a restaurant classified as French and Japanese would have zero (minimal) typicality in all other cuisines, such as Mexican or Californian. This approach had two serious limitations: It could not account for the graded nature of typicality in concepts documented by psychologists, e.g., ref. 5; and it could not measure the typicality of items assigned to a single concept.

In a recent paper, we proposed an approach that overcame these two limitations (6). It consists of using text data to train a probabilistic text classifier which returns the categorization probabilities that the text document is an instance of one or more concepts. The typicality of the text document in a concept is then obtained by taking the logarithm of this categorization probability. In a comparative analysis, we achieved the best performance by “fine-tuning” a text classifier based on BERT on a large amount of training data made of labelled text documents.

The measure we propose in the present article, based on GPT-4, departs from this prior approach in two ways: It does not involve the specification of a classifier at any stage in the construction of the measure, and it does not involve any model training on the research data—it relies on zero-shot learning.

Several recent papers have assessed the classification performance of recent LLMs such as GPT-3 (17), GPT-3.5 Turbo (18–21), FLAN (17, 20) and GPT-4 (21, 22).^*^ The overall picture that emerges from these analyses is that recent LLMs, and in particular GPT-4, are excellent text classifiers even in settings with many candidate categories. Based on this prior work, especially the studies that found high classification performance in which the benchmark was not a ground truth but a categorization judgment by experts, it is reasonable to conjecture that the latest generation of LLMs could be used to produce measures of typicality that achieve a high correlation with human judgment. At the same time, we are not aware of prior work reporting an empirical test of this conjecture.

A recent study used prompts submitted to a Large Language Model (LLM) to construct a continuous measure akin to typicality (23). The authors queried GPT-3 to classify passages from party manifestos (initially used in ref. 24) as Liberal, Conservative, or Neither. GPT-3 returns the probability of the top 5 candidate tokens for text completion. They used this functionality to compute a passage’s ideology score as the model’s estimated “Conservative” probability minus the estimated “Liberal” probability. In terms of the approach we introduced in our previous paper (6), they used GPT-3 to compute the typicality of a passage in the Conservative concept, its typicality in the Liberal concept, and defined its ideological position as the difference between the two typicalities. Our approach to obtaining a measure of typicality from GPT-4 differs in that it does not involve the use of the LLM to classify the text document in a set of discrete categories. Instead, we use GPT-4 to directly generate quantitative typicality measures as responses to our prompts.

## Using GPT-4 to Obtain the Typicality of a Text Document in a Concept

Our approach involves directly asking GPT-4^†^ about the typicality of an object in a focal concept (CONCEPT) based on a short textual description of the object (TEXT) introduced by means of a description (DESCRIPTION).

We use the following prompt, submitted to GPT-4 via the API (words in capital letters are variables in the prompt):

Here’s a DESCRIPTION: “TEXT.” How typical is this DESCRIPTOR of the CONCEPT? Provide your response as a score between 0 and 100 where 0 means “Not typical at all” and 100 means “Extremely typical.”

In this prompt, DESCRIPTOR stands for an informative label that would apply to all objects from which we aim to obtain typicality measures (e.g., “book,” or “tweet.”)

The model output has some level of randomness that can be adjusted with two parameters, “Temperature” (set at its default value of 1, unless otherwise specified) and “Top P” (which controls the sampling of outputs from the posterior distribution on candidate tokens, set at its default value of 1). We leveraged this randomness to obtain 20 independent typicality scores returned by GPT-4 for each text document.

In a small percentage of cases, the model output was not numeric. When this happened, we resubmitted exactly the same prompt, expecting that the randomness inherent in the model output would then produce numeric outputs. We kept submitting the prompt again until we obtained 20 typicality scores for each text document. (See *SI Appendix* for details.)

### Performance Assessment.

We characterize the performance of typicality measures produced with GPT-4 in terms of their correlation with typicality ratings produced by human judges. For each text document, we have several human typicality ratings, and thus, we use the average of the human typicality ratings as the benchmark.

We use two complementary approaches. First, we selected the first of the 20 typicality scores returned by GPT-4 for each document and calculated its Pearson correlation with the average human typicality rating. We repeated this procedure with each of the remaining 19 typicality scores returned by GPT-4 and took the average correlation.

Second, we constructed an aggregate typicality measure by taking the average of the 20 typicality scores returned by GPT-4 for each book description and computed its Pearson correlation with the average human typicality rating. We expected this aggregate measure to perform generally better than a single typicality score since averaging several estimates frequently improves accuracy (25–27).

## Validation with Human Typicality Ratings

We focus on two domains: assessing the typicality of books in literary genres based on short descriptions and the typicality of tweets published by US Congress members in the Democratic and Republican parties.

### Typicality of Books in Literary Genres.

The text documents consist of book descriptions obtained from the Goodreads.com dataset.^‡^ The text descriptions were generally taken from the cover-jacket text or from Amazon.com description (Goodreads.com is owned by Amazon).

#### Test data.

For the Mystery genre, the test data consist of a set of 1,000 book descriptions used in ref. 6.

The text of these book descriptions was taken “as is” from the original data source (we did not process it in any way).

On Goodreads.com, users can assign genre labels to books. The set of book descriptions includes 500 books for which the Mystery label was the modal label. We call these books “Mystery books.” For the remaining 500 books, the modal genre label was not Mystery. We call these books “Non-Mystery books.”

Book descriptions received on average 10 typicality ratings (with a minimum of 7 and maximum of 12) by Prolific Participants who responded to the question “How typical is this book to the Mystery genre,” using a 0 to 100 slider. In the analyses reported below, we use as a benchmark the arithmetic average of the typicality ratings across the coders who rated it. This benchmark is not an absolute ground truth but a measure with imperfect reliability. (See *SI Appendix*, Table S1 for split-half correlations with Spearman–Brown correction).

The test data for analyzing typicality in the Romance genre have the same structure.

#### Measuring typicality with GPT-4.

We directly asked GPT-4 about the typicality of a book in the focal concept based on the text of its description using the prompt copied on the first page of the article (assuming that the focal concept is the Mystery genre). We used exactly the same text for the book descriptions as in the survey employed to obtain human typicality ratings, without any preprocessing.

By following the data collection procedure described in *SI Appendix*, we obtained 20 typicality scores for almost all book descriptions. For one Mystery and three Romance book descriptions, GPT-4 would consistently return nonnumeric responses. These descriptions were removed from the analysis.

The pairwise correlations between typicality scores returned by GPT-4 are very high. In particular, GPT-4 returns typicality scores that are more deterministic than those of human respondents. (See *SI Appendix*, Table S1 for a direct comparison).

#### Results.

##### Single typicality score produced with GPT-4.

The correlation of typicality scores returned by GPT-4 with the average human typicality exceeds .90 for both literary genres, across all books in the test data (Table 1). The correlation is also high among books in the focal category and among books that do not belong to the focal category (higher than 0.70).

**Table 1.** Result summary

		Correlation between typicality measures produced with LLMs and the average human typicality		
Concept	Data	One typicality score returned by GPT-4	Aggregate typicality measure produced with GPT-4	BERT typicality
Book descriptions				
Mystery genre	All books	0.91	0.92	0.87
	Mystery books	0.74	0.76	0.67
	Non-mystery books	0.75	0.77	0.63
Romance genre	All books	0.92	0.92	0.86
	Romance books	0.70	0.73	0.54
	Non-romance books	0.82	0.83	0.72
Tweets				
Democratic party	All tweets	0.88	0.89	0.74
	Democratic party tweets	0.52	0.58	0.30
	Republican party tweets	0.83	0.85	0.57
Republican party	All tweets	0.82	0.85	0.63
	Republican party tweets	0.65	0.71	0.36
	Democratic party tweets	0.72	0.74	0.44

Note: The aggregate typicality measure produced with GPT-4 is the arithmetic average of 20 typicality scores returned by GPT-4 in response to the same prompt. The BERT typicality score is the former state-of-the-art typicality measure.

##### Aggregate typicality measure.

**Fig. 1.**
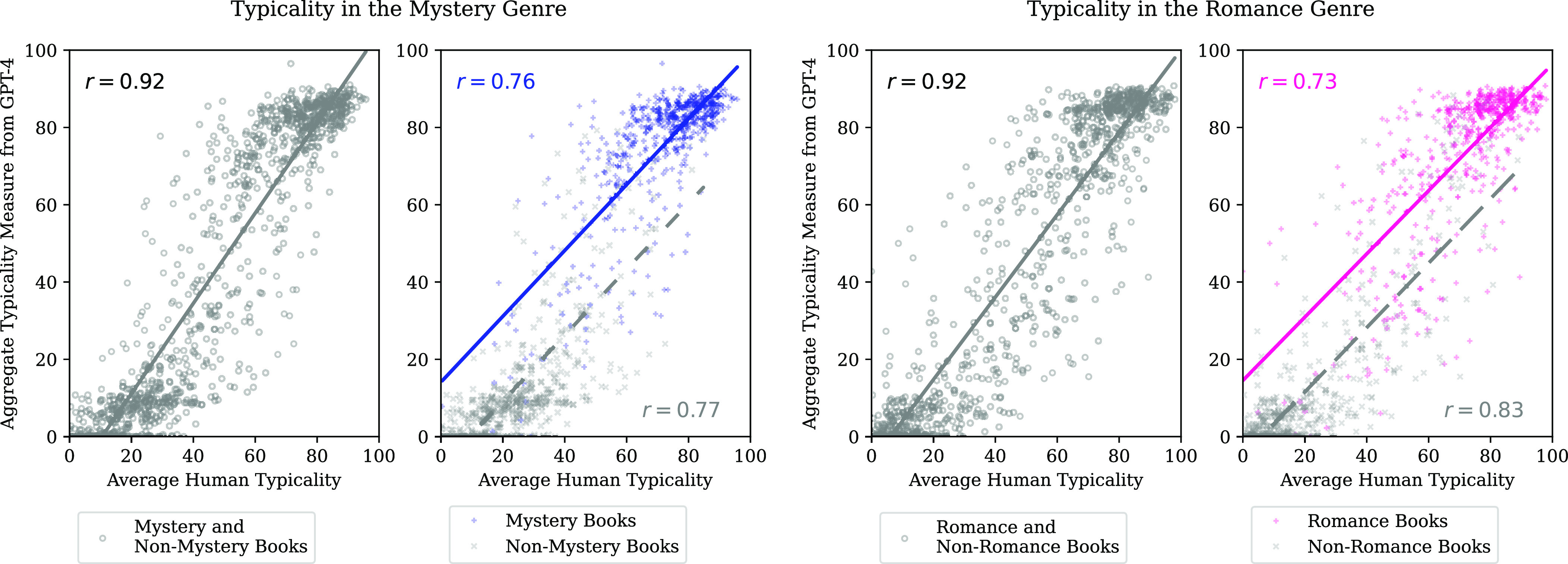
Using GPT-4 to measure the genre typicality of a book based on its description: The aggregate typicality measure produced with GPT-4 is highly correlated with the average of human typicality ratings. *Leftmost panel:* All books in the test data for the Mystery genre. *Center Left panel:* Separate plots for Mystery and Non-Mystery books. *Center Right panel:* All books in the test data for the Romance genre. *Rightmost panel:* Separate plots for Romance and Non-Romance books.

The results are even slightly better with the aggregate typicality measure (Table 1 and Fig. 1). This demonstrates the potential benefit of averaging out the randomness in the model outputs, as frequently observed when averaging predictions of human judges (25, 27). The improvement is very limited, however. This is not surprising because the typicality scores returned by GPT-4 have very high pairwise correlations. The aggregate measure has a reliability greater than .99, making it essentially deterministic (*SI Appendix*, Table S1). This is an advantage for reproducibility, as compared to the use of a single typicality score returned by the LLM.

##### Discussion.

The ability of both the single typicality scores and the aggregate typicality measure to capture within-category differences in typicality means that the measures based on GPT-4 go beyond classification—they capture the internal structure of concepts and categories. In other words, these typicality scores capture the graded nature of typicality documented by psychologists in laboratory studies, e.g., ref. 5.

In ancillary analyses, we assessed the performance of the typicality measures based on GPT-4 obtained by setting up the model to produce more random (temperature equal to 2) and less random outputs (temperature equal to 0).^§^ Results reported in *SI Appendix*, Tables S2 and S3 reveal that the performance of the resulting measures is very similar to that obtained with the default temperature value of 1 (this is especially the case for the aggregate typicality measure).

It is noteworthy that the correlation between the typicality scores returned by GPT-4 and one human typicality rating is consistently larger than the pairwise correlation between human typicality ratings (it also applies to the aggregate typicality measures, *SI Appendix*, Table S1). Further analyses reveal that the aggregate typicality measure produced with GPT-4 is as good a predictor of the typicality ratings of one respondent as the average of 3 to 6 or more human typicality ratings (*SI Appendix*, Table S5). In other words, the model-based measure approximates the ratings of one human participant better than another participant’s ratings.

### Typicality of Tweets in Political Parties.

The text documents consist of tweets published by members of the US Congress, with the focal concepts being the ideological position of the political parties. We aim to measure the typicality of a tweet in the Republican Party and the Democratic Party.

#### Test data.

The test data consist of the texts of a random sample (N=900) of tweets published by members of the 118th US Congress between its opening date (January 3, 2023) and May 1, 2023, who were members of the Democratic Party or Republican Party.^¶^ One half of the tweets were published by Democrats and the other half by Republicans.

The text of the tweet is the “text” field of a tweet object downloaded via the Twitter API. It does not include any metadata such as the username of the user who posted the tweet, number of retweets, etc. It was not preprocessed except for the removal of line breaks (for technical reasons) and the substitution of URLs by the string HIDDEN_URL (the URLs in the text of tweets downloaded with the Twitter API are uninformative strings of characters).

Importantly, the tweets comprising this test set were published after the collection of the GPT-4 pretraining data, which concluded in September 2021. Therefore, cross-contamination between the tweets in the test set and the pretraining data is very unlikely. We obtained between 13 and 18 human typicality ratings for each tweet in each party, with an average of 15 ratings per tweet.

As with the book descriptions, we use as a benchmark the average of the typicality ratings across the participants who rated it. (See data collection details in *SI Appendix*).

#### Measuring typicality with GPT-4.

We employed exactly the same approach as used to analyze the book descriptions, minimally adapting it to the context of tweets. We used the following prompt (assuming for this example that the focal concept is the Republican Party):

Here’s a tweet written by a member of the US Congress: “TEXT”. How typical is this tweet of the Republican Party? Provide your response as a score between 0 and 100 where 0 means “Not typical at all” and 100 means “Extremely typical.”

In this prompt, TEXT stands for the text of the tweet and is exactly the same text as what was used in the survey employed to collect human typicality ratings.

#### Results.

**Fig. 2.**
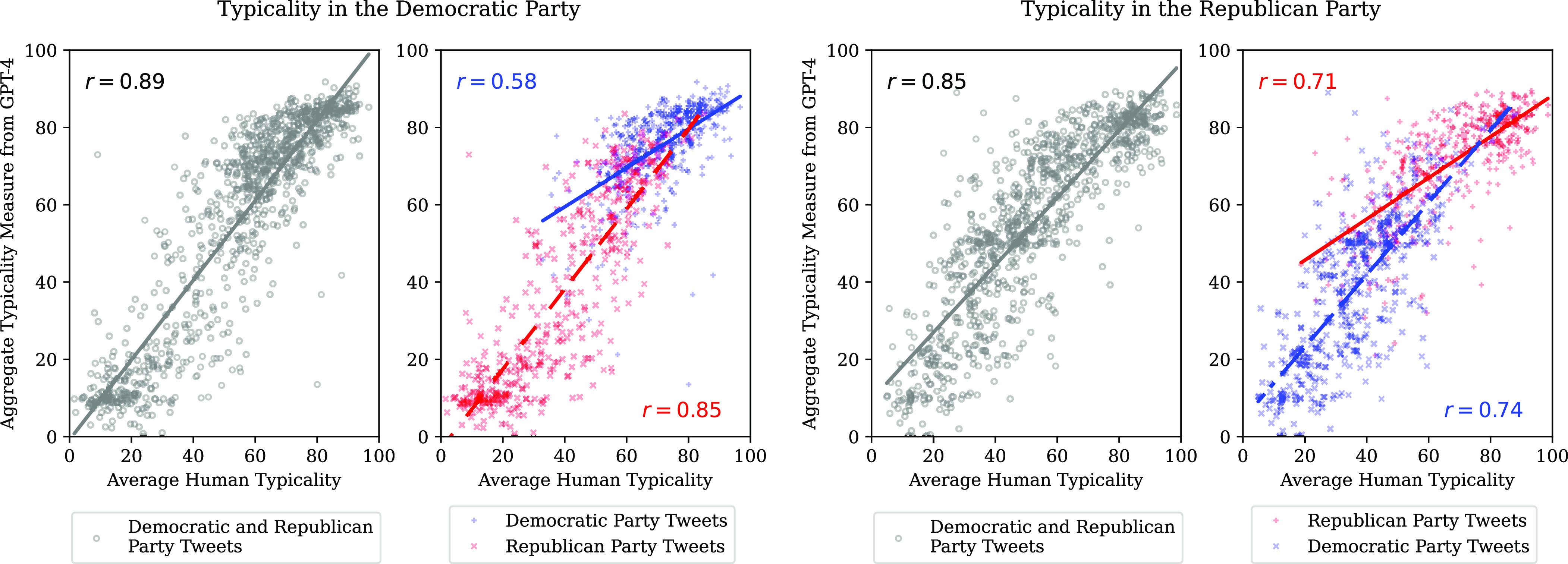
Using GPT-4 to measure the typicality of a tweet in a political party: The aggregate typicality measure produced with GPT-4 is highly correlated with the average of human typicality ratings. *Leftmost panel:* All tweets in the test data for typicality in the Democratic Party. *Center left panel:* Separate plots for tweets by Democratic and Republican Congress members. *Center right panel:* All tweets in the test data for typicality in the Republican Party. *Rightmost panel:* Separate plots for tweets by Democratic and Republican Congress members.

Typicality scores returned by GPT-4 are highly correlated with the responses of the human participants, with values of 0.88 and 0.82 for typicality in the Democratic Party and Republican Party, respectively. This is also the case among tweets published by members of a single political party (Fig. 2 and Table 1). The aggregate measure constructed by averaging the 20 typicality scores performs even better. The improvement is particularly notable in terms of within-category correlations. Examination of the pairwise correlations between typicality scores returned by GPT-4 reveals that it tends to be lower within a set of tweets by members of the focal party. This higher noise in response implies that there is a relatively higher benefit from averaging several scores produced by the LLM.

As with the literary genres, the typicality measures based on GPT-4 approximate the ratings of one human participant better than another participant’s ratings (*SI Appendix*, Table S6).

Both the single typicality scores and the aggregate typicality measure produced with GPT-4 reflect between-tweet differences in human judgments beyond differences in the parties of the politician who published them. In other words, these reflect the internal structure of the political concepts, just as with the literary genres.

## Comparison to the Previous State-of-the-Art (a Measure Obtained by Fine-Tuning BERT)

In ref. 6, we proposed measuring the typicality of an object o in a concept c in terms of the probability that o is a c. This probability is estimated based on the predictions of a text classifier trained on labeled data (of a discrete nature). We hypothesized that model training would enable the text classifier to become sensitive to text features in a manner that mirrors the sensitivity of graded human typicality ratings to text features, even though it is trained on discrete input data. The text classifier uses two distinct components:

1. A language model. The language representation component converts a text document into a vector $x=(x_{1},\ldots ,x_{N})$, where N represents the number of dimensions in the semantic space for text documents.
2. A categorization component that produces categorization probabilities based on the position x of the text document.

A measure of the typicality of a text document in concept c is obtained by taking the logarithm of the categorization probability in c.

### Book Descriptions.

In the comparative analysis reported in ref. 6, the model that achieved the highest correlation with the average of human typicality ratings used BERT as the language model. We fine-tuned the more than 100 million parameters of the BERT text classifier on a training set comprising 680 thousand book descriptions and their genre labels. “Fine-tuning” means that the parameters of the language models and of the categorization component are adjusted by minimizing a classification loss on the training data using deep learning techniques—this optimizes the language representation to the particular task (categorization) and data. We then applied the fine-tuned BERT text classifier to obtain the predicted categorization probabilities in the Mystery genre. What we called “BERT Typicality” is the logarithm of the categorization probability.

This measure performed well. The correlation over all book descriptions and the within-category correlations are very high for both genres (Table 1). Yet, it falls short of the performance of the typicality measures produced with GPT-4.

What is important is not so much the performance difference between the measure produced with GPT-4 and the previous state-of-the-art but the fact that the measure produced with GPT-4 does not involve any training on research data, whereas the previous state-of-the-art involved model fine-tuning on a large training set of 680K text documents. Notably, fine-tuning BERT with a training set of 10K book descriptions instead of the full training set results in a 10% point decrease in the intraclass correlation. Additionally, a typicality measure based on pretrained BERT embeddings and the cosine similarity; thus, involving no training on research data, performs very poorly (*SI Appendix*, Table S4).

### Tweets.

In this context, the probabilistic text classifier predicts the party of the Congress member who published it. The typicality of a tweet in the focal party is obtained by taking the logarithm of the categorization probability produced by the text classifier.

We assembled training data to fine-tune a BERT text classifier and train the other text classifiers analyzed in ref. 6 (these include classifiers that use Bag-of-Words text representations as well as GloVe word embeddings). The training data consist of tweets published by the serving members of the 116th and 117th US Congresses. We obtained these data when we downloaded them via the Twitter API during the first week of May 2023. This encompasses about one million tweets from 537 unique politicians: 270 Democrats and 267 Republicans, spanning January 3, 2019, to January 3, 2023.

Results reported in *SI Appendix*, Table S4 replicate the results obtained with the book descriptions in our previous paper: The best-performing measure (among this set of previously proposed measures) is the one obtained with the BERT model fine-tuned on a large amount of training data (1 million tweets). However, the performance of this typicality measure does not match those based on GPT-4, mirroring the findings with book descriptions. The difference is particularly dramatic among tweets that belong to just one category (e.g., tweets written by members of the Democratic party—see Table 1).

The most important finding is that the measures based on GPT-4 improve on the state-of-the-art even though it does not require any training—we did not train GPT-4 to predict the party of the politicians who published tweets or to predict typicality ratings assembled in a training set.

## Discussion and Conclusion

In this paper, we find that the typicality scores returned by GPT-4 in response to simple prompts achieve a higher correlation with human typicality ratings than previous methods.

The groundbreaking nature of this finding lies in the fact that this performance is achieved without training the LLM on the research data. This breakthrough essentially eliminates the trade-off between the size of the training data and the precision of the model-based measure. It overcomes the limitations of current approaches to measuring typicality with NLP-based techniques. This makes the practical applicability of model-based typicality measures much broader, because assembling a training set frequently requires employing research assistants to manually code thousands or tens of thousands of text documents even when pretrained LLMs such as BERT are used, e.g., refs. 30 and 31. Therefore, the approach based on GPT-4 dramatically reduces the financial and logistical costs of obtaining model-based typicality measures.

Our findings suggest that the semantic space constructed by GPT-4 closely matches that of human judges. This opens the door to constructing other measures useful to social scientists that go beyond classification in a discrete set of categories (or “tagging”) such as ambiguity, diversity, extremity, and polarization.

A frequently heard concern regarding the outputs of LLMs is that they lack interpretability. This concern is valid in some use cases, but we do not think it is a critical issue in many other use cases. The reason is that the interpretability concerns affecting the outputs of LLMs are similar to those affecting human coding, even though we often demand more of machines than of humans. There is much evidence in psychology that humans are quite poor at explaining how they produce their own judgments, e.g., ref. 32. For instance, asking research assistants or experts to explain their rating process for the typicality of text documents within a concept might not offer much insight into the judgment’s genesis. In other words, human coding is unlikely to be more interpretable than the outputs of LLMs. In settings in which researchers are satisfied with human coding, they should probably be equally satisfied with LLM-based measures, provided they have collected test data from human judges and verified that the model-based measures have a sufficiently high correlation with the measures provided by human judges on the test data.

The results presented in this paper align with the emerging sentiment about the advantages of using the most recent LLMs. Yet, we conclude with a few words of caution regarding the use of LLMs as measurement devices for social science. The strengths and weaknesses of LLMs come from their pretraining.

Pretraining allows the model to learn semantic associations present in text data written by humans. The models are thus bound to reproduce these associations in the semantic space they construct and in the text they produce in response to queries (33). If the pretraining data contains erroneous semantic associations or associations that correspond to prejudice against particular social groups, ideas, or ideology, these will influence the measures produced by LLMs. It is thus crucial to validate measures based on the outputs of LLMs for each use case. In ancillary analyses, we found that the performance achieved by recent pretrained LLMs that were released between BERT and GPT-4 (GPT-3, GPT-3.5, and GPT-3 embeddings) falls short of that achieved by GPT-4 and is sometimes very poor, e.g., GPT-3; (*SI Appendix*, Tables S7 and S8). Validation of the model-based measure is thus necessary in each case, at least until the research community identifies general principles to guide end-users in the identification of domains where validation is necessary and where prior research is sufficient to trust the model-based measures. Whether it is at all possible to develop such principles is an open question.

## Supplementary Material

Appendix 01 (PDF)

## Data Availability

Anonymized CSV files have been deposited in OSF (https://doi.org/10.17605/OSF.IO/EMHWC) (34).

## Footnotes

^*^Some of these papers have also analyzed other tasks such as mathematical reasoning or question answering (21).

^†^We used the most recently published GPT model at the time of this research: GPT-4 (gpt-4-0613) (Snapshot from June 13, 2023).

^‡^Made publicly available by UCSD at https://sites.google.com/eng.ucsd.edu/ucsdbookgraph/home, (28, 29).

^§^These are the minimal and maximal values for the temperature parameter, according to openAI’s API reference.

^¶^These consist of around 91 thousand tweets. We downloaded the tweets on May 2, 2023. We used the list of congress members and Twitter usernames made available by https://github.com/unitedstates/congress-legislators. We consider only original tweets of more than 2 tokens (not replies, retweets, or quotes of tweets).
