# Supplementary material for: Uncovering the semantics of concepts using GPT-4
Source: Proc Natl Acad Sci U S A. 2023 Nov 30;120(49):e2309350120. doi: 10.1073/pnas.2309350120 (PMC10710071; doi:10.1073/pnas.2309350120)
Supplement: Supplementary file 1 — Appendix 01 (PDF) [file pnas.2309350120.sapp.pdf]

Supporting Information for

Uncovering the Semantics of Concepts Using GPT-4

Gaël Le Mens, Balázs Kovács, Michael T. Hannan and Guillem Pros

Gaël Le Mens.
E-mail: gael.le-mens@upf.edu

This PDF file includes:

Supporting text
Figs. S1 to S2
Tables S1 to S8
SI References

Supporting Information Text

1. Methodological Details

Data and scripts are available at https://osf.io/emhwc.

Details on API implementation and the handling of non-numeric responses by GPT-4. In our implementation, we used the ChatCompletion command of the OpenAI API to submit the prompts to GPT-4 and collect the outputs.

We used the default parameter values, except for parameter ‘n’, which we set to 25. This allowed us to obtain 25 independent responses to the same prompt. In a small percentage of cases, one or several of the responses did not contain a number, which was inconsistent with the instructions given in the prompt. If at least 20 of them were numerical scores, we kept them and dropped the rest from the analysis. If we obtained fewer than 20 typicality scores (this was the case for 2 book descriptions in the Mystery data and for 11 of them in the Romance data), we requested another 25 independent responses, and so on, until a maximum of 125 GPT-4 responses).

At the end of this process, we obtained 20 typicality scores for almost all text documents in our four datasets. The text documents with fewer than 20 typicality scores were dropped from the analysis.

Ancillary investigations reveal two possible reasons for the failure to obtain the desired number of typicality scores. In some cases, visual inspection of the text documents reveals that they did not contain information that could be used to make a reasonable typicality judgment (our test datasets were random samples of larger datasets described in the body of the paper, and we did not visually inspect the book descriptions and tweets before inclusion in the test data). This is what happened for 4 book descriptions for which we did not obtain the minimal number of typicality scores. In some cases, visual inspection of the GPT-4 response suggests that the prompt triggered a ‘rule-based’ response that prevented GPT-4 from providing a response.

IDs of the text documents with less than 20 numeric responses:

- Mystery book concept data: 18507144.
- Romance book concept data: 13355203, 1185608, 2880995.
- Democratic tweet concept data: 188
- Republican tweet concept data: 68, 176, 710.

Survey of Book Typicality Ratings. The survey was conducted for our previous paper (1). Here we copy the survey prompt to illustrate the Goodreads book descriptions and also the prompt used in the survey.

Fig. S1. Example of typicality rating display.

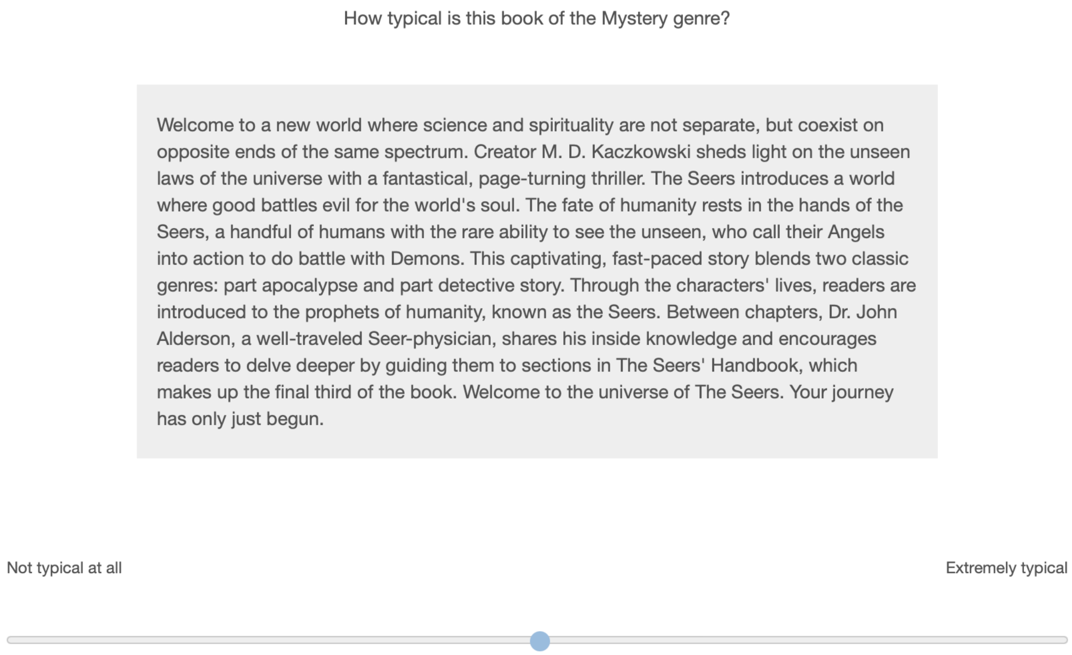

Survey of Tweet Typicality Ratings. The online survey was administered in the second week of May 2023. 903 Prolific participants (U.S. residents, average age of 41 years, 57% male, 41% female, 2% others) were randomly assigned to one of two "party conditions." Half of the participants provided typicality ratings of tweets in the Democratic Party. The other half did so for the Republican Party. The 900 tweets in the test set were rated by participants in the two conditions. We constructed 30 sets of 30 tweets, composed of 15 tweets from each party. Participants were randomly allocated to one of these sets. We obtained between 13 and 18 typicality ratings for each tweet (in each party), with an average of 15 ratings per tweet. To provide their typicality ratings, participants responded to the question (assuming for this example that the focal concept is the Democratic Party) "How typical is this tweet to the Democratic Party?" using a 0 to 100 slider (centered at 50 when the page appears on the screen). Importantly, participants were not provided with any information about the author of the tweet — just the text of the tweet. Therefore, they were not informed of whether the tweet was written by a Democrat or a Republican. To ensure response quality, we screened Prolific participants based on the following criteria:

- Fluent languages includes English;
- Approval Rate: Minimum 95%;
- Country of Residence: US;
- Political Spectrum (US): they should have provided a response to this question (the response could be anyone of {Conservative, Moderate, Liberal, Other}),
- U.S. Political Affiliation: they should have provided a response to this question (the response could be anyone of {Democrat, Republican, Independent, Other, None}).

The last two items were included to increase the likelihood that participants have some knowledge of US politics. After providing informed consent, participants were asked

> Please take a moment to write about your expectations regarding tweets by politicians of the [Democratic Party / Republican Party]. You could write about possible topics addressed by such tweets, and or opinions you expect the authors to have about these topics (min. response length: 100 characters).

On the next page, they were provided with short instructions about the typicality rating task.

> For each of the 30 tweets, you will be asked the following question: How typical is this tweet of the [Democratic Party / Republican Party]? You will report your response using a continuous slider that goes from 'Not typical at all' (0) to 'Extremely typical' (100). There is no right or wrong answer. We are interested in your subjective opinion. Note that the slider will appear on the screen 6 seconds after the text of the tweet.

Then they looped through the 30 tweets and provided their typicality ratings for each of them. See Figure S2 for an example of a typicality rating display.

Finally, the study concluded with a short demographic survey, and some questions about their political orientation, their political opinions and their frequency of use of Twitter.

Fig. S2. Example of typicality rating display.

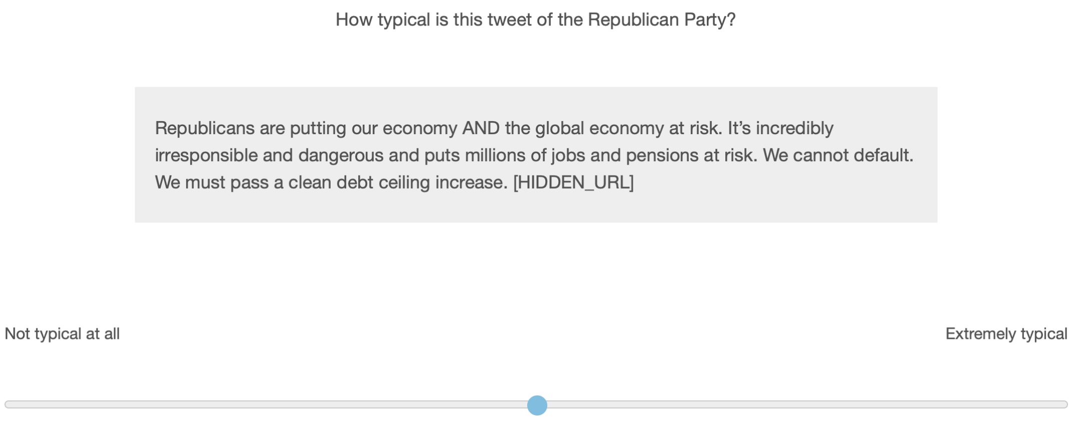

2. Additional Results 1: Pairwise Correlations, reliability, and replication of table 1 with different values of the temperature parameter

Table S1. Pairwise correlations and reliability of human typicality ratings and typicality scores returned by GPT-4.

			Human typicality ratings		Typicality scores returned by GPT-4	
	Concept	Data	Pairwise correlation	Reliability of the average	Pairwise correlation	Reliability of the average
Book descriptions	Mystery Genre	All books	.53	.92	.98	.999
		Mystery books	.27	.78	.92	.996
		Non-Mystery books	.27	.78	.96	.998
	Romance Genre	All books	.64	.95	.98	.999
		Romance books	.31	.82	.93	.996
		Non-Romance books	.44	.88	.97	.998
Tweets	Democratic Party	All tweets	.47	.93	.96	.998
		Democratic Party tweets	.18	.77	.79	.987
		Republican Party tweets	.35	.89	.96	.998
	Republican Party	All tweets	.44	.92	.95	.997
		Republican Party tweets	.33	.88	.85	.991
		Democratic Party tweets	.23	.82	.92	.996

Note: The reliability of the average of human ratings was computed using split-half correlations with Spearman-Brown correction, because not all text documents had the same number of human ratings. The reliability of the aggregate typicality measure (average of the 20 typicality scores returned by GPT-4) was computed using Cronbach's alpha.

Table S2. Pairwise correlations and reliability of the average typicality scores returned by GPT-4 for different temperature values. (temperature = 0: less randomness in output, temperature = 1: default value, temperature = 2: maximal randomness in output.)

			Typicality scores returned by GPT-4		Typicality scores returned by GPT-4		Typicality scores returned by GPT-4	
	Temperature		1	1	0	0	2	2
	Concept	Data	Pairwise correlation	Reliability of the average	Pairwise correlation	Reliability of the average	Pairwise correlation	Reliability of the average
Book descriptions	Mystery Genre	All books	.98	.999	.999	1.000	.96	.998
		Mystery books	.92	.996	.994	1.000	.84	.991
		Non-Mystery books	.96	.998	.997	1.000	.92	.996
	Romance Genre	All books	.98	.999	.999	1.000	.97	.998
		Romance books	.93	.996	.995	1.000	.85	.991
		Non-Romance books	.97	.998	.998	1.000	.94	.997
Tweets	Democratic Party	All tweets	.96	.998	.998	1.000	.92	.995
		Democratic Party tweets	.78	.986	.990	.999	.61	.968
		Republican Party tweets	.95	.998	.997	1.000	.90	.995
	Republican Party	All tweets	.95	.997	.997	1.000	.88	.993
		Republican Party tweets	.85	.991	.991	1.000	.70	.979
		Democratic Party tweets	.92	.996	.996	1.000	.84	.990

Note: The reliability of the aggregate typicality measure (average of the 20 typicality scores returned by GPT-4) was computed using Cronbach's alpha.

Table S3. Correlation between the typicality measures based on GPT-4 and the average human typicality with different levels of the temperature parameter (temperature = 0: less randomness in output, temperature = 1: default value, temperature = 2: maximal randomness in output.)

			Correlation between typicality measures produced with GPT-4 and the average human typicality					
			One typicality score returned by GPT-4	Aggregate typicality measure produced with GPT-4	One typicality score returned by GPT-4	Aggregate typicality measure produced with GPT-4	One typicality score returned by GPT-4	Aggregate typicality measure produced with GPT-4
	Temperature		1	1	0	0	2	2
	Concept	Data						
Book descriptions	Mystery Genre	All books	.91	.92	.92	.92	.90	.92
		Mystery books	.74	.76	.75	.76	.71	.77
		Non-Mystery books	.75	.77	.76	.76	.73	.76
	Romance Genre	All books	.92	.92	.92	.92	.91	.92
		Romance books	.70	.73	.72	.72	.68	.73
		Non-Romance books	.82	.83	.82	.82	.80	.83
Tweets	Democratic Party	All tweets	.88	.89	.89	.89	.89	.86
		Democratic Party tweets	.52	.58	.56	.56	.57	.45
		Republican Party tweets	.83	.85	.84	.84	.85	.81
	Republican Party	All tweets	.82	.85	.84	.84	.79	.84
		Republican Party tweets	.65	.71	.74	.74	.68	.74
		Democratic Party tweets	.72	.74	.69	.69	.59	.70

Note: The aggregate typicality measure produced with GPT-4 is the arithmetic average of 20 typicality scores returned by GPT-4 in response to the same prompt.

3. Additional Results 2: Performance of the Model-Based Typicality Measures Analyzed in (1) on the Tweet Data

In the body of the paper, we focused on comparing the performance of the typicality measures produced by *GPT-4* and *BERT*. This comparison is justified in the context of the book descriptions, because prior work found that *BERT* had the highest correlation with the average human typicality rating. Here, we report analyses that show that the same applies to the typicality of tweets in the two main US parties. Table S4 reports a replication of the analyses performed on the book description data by (1).* What is important for the present paper is that the highest performing measures are the typicalities obtained from fine-tuning BERT. Overall, the performance ordering of the previous measures is extremely similar in this setting to the ordering that was obtained with the book description data, and therefore we do not discuss it further here (we direct interested readers to the result section of (1)).

Table S4. Typicality of tweets in the Democratic Party and the Republican Party: Performance of previous model-based typicality measures. (Replication of the comparative analysis in (1) with the tweet data).

Typicality in the Democratic Party

			Correlations between model-based typicalities and human typicality ratings			Model Training					
		Typicality Measure	All tweets	Democratic Party tweets	Republican Party tweets	Training Sample	Fine-tuning the language representation	Training a probabilistic classifier	Language representation	Sensitivity to word order	Similarity between text document and concept
LLM	BERT	BERT fine-tuned / cat. proba. (*BERT Typicality*)	.74	.30	.57	1M tweets	Yes	Yes	fine-tuned BERT	Yes	Log(Cat. Prob.)
LLM	BERT	BERT fine-tuned / corr. with. prototype	.75	.31	.55	1M tweets	Yes	Yes	fine-tuned BERT	Yes	Cosine
LLM	BERT	BERT pre-trained / cat. proba.	.52	.35	.30	1M tweets	No	Yes	pre-trained BERT	Yes	Log(Cat. Prob.)
LLM	BERT	BERT pre-trained / corr. with. prototype	.07	.13	.11	None	No	No	pre-trained BERT	Yes	Cosine
Methods not sensitive to word order	GloVe Word Embeddings	GloVe fine-tuned / cat. proba.	.60	.31	.36	1M tweets	Yes	Yes	fine-tuned GloVe	No	Log(Cat. Prob.)
Methods not sensitive to word order	GloVe Word Embeddings	GloVe fine-tuned / corr. with. prototype	.50	.26	.27	1M tweets	Yes	Yes	fine-tuned GloVe	No	Cosine
Methods not sensitive to word order	GloVe Word Embeddings	GloVe pre-trained / cat. proba.	.49	.22	.32	1M tweets	No	Yes	pre-trained GloVe	No	Log(Cat. Prob.)
Methods not sensitive to word order	GloVe Word Embeddings	GloVe pre-trained / corr. with. prototype	.13	.20	.02	None	No	No	pre-trained GloVe	No	Cosine
Methods not sensitive to word order	Word Frequencies	Word Frequencies / cat. proba.	.59	.30	.44	1M tweets	No	Yes	BoW Term Frequencies	No	Log(Cat. Prob.)
Methods not sensitive to word order	Word Frequencies	TF-IDF / cat. proba	.62	.36	.46	1M tweets	No	Yes	BoW TF-IDF	No	Log(Cat. Prob.)
Methods not sensitive to word order	Word Frequencies	TF-IDF / corr. with. prototype	.22	.09	.21	None	No	No	BoW TF-IDF	No	Cosine

Typicality in the Republican Party

			Correlations between model-based typicalities and human typicality ratings			Model Training					
		Typicality Measure	All tweets	Democratic Party tweets	Republican Party tweets	Training Sample	Fine-tuning the language representation	Training a probabilistic classifier	Language representation	Sensitivity to word order	Similarity between text document and concept
LLM	BERT	BERT fine-tuned / cat. proba. (*BERT Typicality*)	.63	.44	.36	1M tweets	Yes	Yes	fine-tuned BERT	Yes	Log(Cat. Prob.)
LLM	BERT	BERT fine-tuned / corr. with. prototype	.63	.26	.40	1M tweets	Yes	Yes	fine-tuned BERT	Yes	Cosine
LLM	BERT	BERT pre-trained / cat. proba.	.50	.23	.32	1M tweets	No	Yes	pre-trained BERT	Yes	Log(Cat. Prob.)
LLM	BERT	BERT pre-trained / corr. with. prototype	.05	-.01	.01	None	No	No	pre-trained BERT	Yes	Cosine
Methods not sensitive to word order	GloVe Word Embeddings	GloVe fine-tuned / cat. proba.	.56	.33	.35	1M tweets	Yes	Yes	fine-tuned GloVe	No	Log(Cat. Prob.)
Methods not sensitive to word order	GloVe Word Embeddings	GloVe fine-tuned / corr. with. prototype	.37	.24	.27	1M tweets	Yes	Yes	fine-tuned GloVe	No	Cosine
Methods not sensitive to word order	GloVe Word Embeddings	GloVe pre-trained / cat. proba.	.48	.36	.19	1M tweets	No	Yes	pre-trained GloVe	No	Log(Cat. Prob.)
Methods not sensitive to word order	GloVe Word Embeddings	GloVe pre-trained / corr. with. prototype	.02	.19	.01	None	No	No	pre-trained GloVe	No	Cosine
Methods not sensitive to word order	Word Frequencies	Word Frequencies / cat. proba.	.54	.32	.37	1M tweets	No	Yes	BoW Term Frequencies	No	Log(Cat. Prob.)
Methods not sensitive to word order	Word Frequencies	TF-IDF / cat. proba	.60	.49	.33	1M tweets	No	Yes	BoW TF-IDF	No	Log(Cat. Prob.)
Methods not sensitive to word order	Word Frequencies	TF-IDF / corr. with. prototype	.04	.09	.09	None	No	No	BoW TF-IDF	No	Cosine

*The measures based on label proportions do not have any equivalent to this context; the same applies to the measure based on training a text classifier with 36 target categories.

4. Additional Results 3: Predicting the Typicality Ratings of One Human Respondent: ENO scores

One existing characterization of the predictive ability of models focuses on predicting the behavior of one human respondent (2). It asks how many observations of other human participants are necessary to make a prediction as good as the model. The resulting number is the model's equivalent number of observations (ENO).

Here, we predict the typicality ratings of one human participant based on the average of N typicality ratings of other participants. We compute the Pearson correlation between these two quantities. The resulting correlation will depend on the specific ratings selected as criterion and predictor variables. So, for each text document, we randomly sample one human rating as the criterion variable and treat the remaining ratings as potential predictors. We repeat this procedure 1,000 times and report the average correlation in Tables S5 and S6. We do this for values of N that range from 1 to the maximum possible (the number of human typicality ratings minus 1).

These tables also report the Pearson correlation between the two typicality measures based on GPT-4 (single typicality score and aggregate typicality measure based on the 20 typicality scores) and one human typicality rating.

The ENO score for the typicality measures based on GPT-4 is equal to 2 or better (it reaches 7 in some cases). We computed the ENO score for BERT on the tweet data. It is generally equal to 1 (and equal to 2 in one case).

Table S5. Book data: Predicting one human typicality rating with other human typicality ratings and with typicality measures based on GPT-4.

		Typicality in Mystery Genre			Typicality in Romance Genre		
		All books	Mystery books	Non-Mystery books	All books	Romance books	Non-Romance books
Predictor		Average correlation between one human typicality rating and predictor					
Average of N human typicality ratings with	N = 1	.53	.27	.26	.64	.31	.44
	N = 2	.61	.34	.33	.71	.39	.52
	N = 3	.64	.38	.37	.74	**.43**	.56
	N = 4	.66	.40	.40	.75	<u>.45</u>	<u>**.58**</u>
	N = 5	.67	.42	.41	<u>**.76**</u>	.47	.59
	N = 6	<u>**.68**</u>	<u>**.43**</u>	<u>**.43**</u>	.77	.48	.60
One typicality score returned by GPT-4		.69	.43	.44	.76	.43	.58
Aggregate typicality measure produced with GPT-4		.70	.45	.45	.76	.45	.58
		ENO score					
One typicality score returned by GPT-4		≥6	6	≥6	5	3	4
Aggregate typicality measure produced with GPT-4		≥6	≥6	≥6	5	4	4

Note: The ENO is the model's equivalent number of observations by other human respondents to make a prediction as accurate as the model. The book description with the smallest number of human typicality ratings had 7 ratings. Bold values correspond to the ENO score of one typicality score returned by GPT-4, and underlined values correspond to the ENO score of the aggregate typicality measure produced with GPT-4.

Table S6. Tweet data: Predicting one human typicality rating with other human typicality ratings and with typicality measures based on GPT-4.

Predictor		Typicality in Democratic Party			Typicality in Republican Party		
		All tweets	Democratic Party tweets	Republican Party tweets	All tweets	Democratic Party tweets	Republican Party tweets
		Average correlation between one human typicality rating and predictor					
Average of N human typicality ratings with	N = 1	.47	*.18*	*.35*	*.44*	*.23*	*.33*
	N = 2	*.55*	**.24**	.43	.51	.29	**.40**
	N = 3	.58	<u>.27</u>	.47	.55	.33	<u>.44</u>
	N = 4	.60	<u>.29</u>	.49	**<u>.57</u>**	.35	.47
	N = 5	**.62**	.31	.51	<u>.59</u>	.37	.49
	N = 6	.63	.32	**.52**	.60	**.38**	.50
	N = 7	.63	.34	<u>.53</u>	.61	<u>.39</u>	.51
	N = 8	<u>.64</u>	.34	<u>.53</u>	.61	.40	.51
	N = 9	<u>.64</u>	.35	.54	.62	.41	.52
	N = 10	.65	.36	.55	.62	.41	.52
	N = 11	.65	.36	.55	.62	.42	.53
	N = 12	.65	.37	.55	.63	.42	.53
One typicality score returned by GPT-4		.62	.25	.52	.57	.38	.40
Aggregate typicality measure produced with GPT-4		.64	.28	.53	.58	.39	.43
BERT typicality		.53	.15	.36	.44	.23	.22
		ENO score					
One typicality score returned by GPT-4		5	2	6	4	6	2
Aggregate typicality measure produced with GPT-4		8-9	3-4	7-8	4-5	7	3
BERT typicality		2	<1	1	1	1	<1

Note: The ENO is the model's equivalent number of observations by other human respondents to make a prediction as accurate as the model. The tweet with the smallest number of human typicality ratings had 12 ratings. Bold values correspond to the ENO score of one typicality score returned by GPT-4, underlined values correspond to the ENO score of the aggregate typicality measure produced with GPT-4, and italicized values correspond to the ENO score of the BERT typicality.

5. Additional Results 4: Comparing Typicality Measures based on GPT-4 Typicality with Measures Based on Other Recent LLMs

We use other LLMs of the GPT family of models to obtain typicality measures.

GPT Text Embeddings. A frequently used approach to measuring typicality defines the concept prototype as the average position of instances of this concept in semantic space, and takes the cosine similarity between the position of a text document and the prototype as its typicality (e.g., 3–5). Text representations consist of Bag-of-Words TF-IDF ("Term Frequency–Inverse Document Frequency," 6) or pre-trained word embeddings, generally seen as a cutting-edge method in social science (here implemented with GloVe embeddings (7)). In (1), we reported the performance of measures based on these representations on the book description data. We replicated these analyses with the tweet data. Results reported in (1) (book descriptions) and in Table S4 (tweets) show that, with these two representations, the correlation between model-based typicality measures and the average human typicality is *at best moderate* $(r < .3)$.

We applied the approach described in the previous paragraph to a much more recent embedding model: a text embedding model of the same generation as GPT-3 (the LLM that powered ChatGPT when it was first released): text-embedding-ada-002, released December 15, 2022. Just like BERT, this model transforms text documents in vectors. Results reported in Table S7 show that the performance of the cosine similarity measure obtained with this LLM, to measure the typicality of book descriptions in literary genres, is much better than that obtained with the pre-trained BERT model or the cosine similarity and simpler representations like Bag-of-Words TF-IDF and GloVe Embeddings (see (1)). This indicates that the semantic space at the core of the model reflects human judgments of genre typicality (in the Mystery and Romance genres) much better than do the earlier generations of machine text representation.

At the same time, the performance of this measure with the tweet data is very poor (Table S8). This implies that the semantic space at the core of this model does not have a representation of the concepts Democratic Party and Republican Party that matches those of the participants in our survey. This finding should serve as a warning call to researchers who feel tempted to use recent Large Language Models (LLMs) to construct similarity measures without validating them on empirical data.

To assess whether this text representation could nevertheless be leveraged to produce a typicality measure that corresponds well with human typicality ratings, we applied the method we advocated in (1): we trained a text classifier based on this representation using the 1 million tweet training dataset.[†] The resulting performance is fairly good, it manages to surpass that of the typicality based on the fine-tuned BERT model in some instances (e.g., within category correlations for tweets written by Democratic Congress members). This indicates that model training identified the dimensions in embedding space that matter for typicality judgments. Yet, the performance of this measure remains quite far from that of the measures produced with GPT-4, even though it involved training a model on a large training data set.

Text Completion with Pre-Trained GPT-3 and GPT-3.5. We adapted the prompt we used with GPT-4 for use with models of the previous generation: GPT-3 and GPT-3.5. The models we consider have not been specifically designed to work as chatbots, but instead to complete text that has been started by the user. Therefore, we adapted the prompt by adding the beginning of the response at the end of the prompt. Assuming for this example that we aim to measure the typicality of a tweet in the Republican Party, we used:

> Here's a tweet written by a member of the US Congress: 'TEXT'. How typical is this tweet of the Republican Party? Provide your response as a score between 0 and 100 where 0 means 'Not typical at all' and 100 means 'Extremely typical'. The tweet typicality score is:

We used a model of the GPT-3 family (text-curie-001, released in 2019) and a model of the GPT-3.5 family (text-davinci-003, released in November 2022). With both models, we aimed to obtain one typicality rating per text documents (we re-submitted the same prompt up to 20 times if we did not obtain a numeric response). [‡] Results reported in Tables S7 and S8 indicate that, whereas the typicality scores provided by the recent GPT-3.5 model have a high correlation with the average human typicality (roughly matching that of the typicality measure constructed with fine-tuned BERT), this is not the case for the typicality ratings provided by the GPT-3 model. To the contrary, these have an extremely low correlation with human judgment. The correlation is even (slightly) negative in the case of book descriptions. And with the tweets, more than 90% of the responses were a typicality score of "50."

This finding demonstrates that researchers should apply caution when using LLMs to construct typicality measures. In some settings, they fail to match human judgments, and relying on such measures in downstream analyses would lead to results of questionable empirical validity. Empirical validation is necessary to avoid falling into such pitfalls.

[†] We used a random-forest text classifier.

[‡] We also tried to use the two most capable original GPT-3 models (curie and davinci) but they did not respond to completion requests with numbers and thus the outputs were unusable.

Table S7. Typicality of books in the Mystery and Romance genres: Comparing the performance of the typicality measures produced with GPT-4, and measures based on BERT and other LLMs more recent than BERT.

**Typicality in the Mystery Genre**		Correlations between model-based typicalities and human typicality ratings			Model Training				
Release year	Typicality Measure	All books	Mystery books	Non-Mystery books	Training Sample	Fine-tuning the language representation	Training a probabilistic classifier	Language representation	Similarity between text document and concept
2023-06	GPT-4 Aggregate Typicality	.92	.76	.77	None	No	No	gpt-4-0613	Av. typicality score
	GPT-4 Typicality Score	.91	.74	.75					Typicality score
2018-10	BERT fine-tuned / cat. Proba.	.87	.67	.63	680K	Yes	Yes	fine-tuned bert-base-uncased	Log(Cat. Prob.)
	BERT pre-trained / corr. with. prototype	.07	.03	.07	None	No	No	bert-base-uncased	Cosine
2022-12	ada2 pre-trained / cat.proba	.82	.63	.49	680K	No	Yes	text-embedding-ada-002	Log(Cat. Prob.)
	ada2 pre-trained / corr. with. prototype	.76	.51	.61	None		No		Cosine
2022-11	Pre-trained GPT-3.5 Completion	.81	.57	.65	None	No	No	text-davinci-003	Typicality score
2019	Pre-trained GPT-3 Completion	-.07	.00	-.17	None	No	No	text-curie-001	Typicality score

**Typicality in the Romance Genre**		Correlations between model-based typicalities and human typicality ratings			Model Training				
Release year	Typicality Measure	All books	Romance books	Non-Romance books	Training Sample	Fine-tuning the language representation	Training a probabilistic classifier	Language representation	Similarity between text document and concept
2023-06	GPT-4 Aggregate Typicality	.92	.73	.83	None	No	No	gpt-4-0613	Av. typicality score
	GPT-4 Typicality Score	.92	.70	.82					Typicality score
2018-10	BERT fine-tuned / cat. Proba.	.86	.54	.72	680K	Yes	Yes	fine-tuned bert-base-uncased	Log(Cat. Prob.)
	BERT pre-trained / corr. with. prototype	.07	.11	.04	None	No	No	bert-base-uncased	Cosine
2022-12	ada2 pre-trained / cat.proba	.86	.59	.70	680K	No	Yes	text-embedding-ada-002	Log(Cat. Prob.)
	ada2 pre-trained / corr. with. prototype	.79	.47	.65	None		No		Cosine
2022-11	Pre-trained GPT-3.5 Completion	.88	.53	.76	None	No	No	text-davinci-003	Typicality score
2019	Pre-trained GPT-3 Completion	-.27	-.07	-.16	None	No	No	text-curie-001	Typicality score

Table S8. Typicality of tweets in the Democratic Party and the Republican Party: Comparing the performance of the typicality measures produced with GPT-4, and measures based on BERT and other LLMs more recent than BERT.

**Typicality in the Democratic Party**		Correlations between model-based typicalities and human typicality ratings			Model Training				
Release year	Typicality Measure	All tweets	Democratic Party tweets	Republican Party tweets	Training Sample	Fine-tuning the language representation	Training a probabilistic classifier	Language representation	Similarity between text document and concept
2023-06	GPT-4 Aggregate Typicality	.89	.58	.85	None	No	No	`gpt-4-0613`	Av. typicality score
	GPT-4 Typicality Score	.88	.52	.83					Typicality score
2018-10	BERT fine-tuned / cat. Proba.	.74	.30	.57	1M tweets	Yes	Yes	fine-tuned `bert-base-uncased`	Log(Cat. Prob.)
	BERT pre-trained / corr. with. prototype	.07	.13	.11	None	No	No	`bert-base-uncased`	Cosine
2022-12	ada2 pre-trained / cat.proba	.65	.42	.48	1M tweets	No	Yes	`text-embedding-ada-002`	Log(Cat. Prob.)
	ada2 pre-trained / corr. with. prototype	.11	.16	.13	None		No		Cosine
2022-11	Pre-trained GPT-3.5 Completion	.59	.36	.46	None	No	No	`text-davinci-003`	Typicality score
2019	Pre-trained GPT-3 Completion	.13	.06	.13	None	No	No	`text-curie-001`	Typicality score
**Typicality in the Republican Party**		Correlations between model-based typicalities and human typicality ratings			Model Training				
Release year	Typicality Measure	All tweets	Democratic Party tweets	Republican Party tweets	Training Sample	Fine-tuning the language representation	Training a probabilistic classifier	Language representation	Similarity between text document and concept
2023-06	GPT-4 Aggregate Typicality	.85	.74	.71	None	No	No	`gpt-4-0613`	Av. typicality score
	GPT-4 Typicality Score	.82	.72	.65					Typicality score
2018-10	BERT fine-tuned / cat. Proba.	.63	.44	.36	1M tweets	Yes	Yes	fine-tuned `bert-base-uncased`	Log(Cat. Prob.)
	BERT pre-trained / corr. with. prototype	.05	-.01	.01	None	No	No	`bert-base-uncased`	Cosine
2022-12	ada2 pre-trained / cat.proba	.67	.40	.55	1M tweets	No	Yes	`text-embedding-ada-002`	Log(Cat. Prob.)
	ada2 pre-trained / corr. with. prototype	.14	.01	.07	None		No		Cosine
2022-11	Pre-trained GPT-3.5 Completion	.62	.57	.34	None	No	No	`text-davinci-003`	Typicality score
2019	Pre-trained GPT-3 Completion	.01	.10	-.01	None	No	No	`text-curie-001`	Typicality score

References

1. G Le Mens, B Kovács, MT Hannan, G Pros Rius, Using machine learning to uncover the semantics of concepts: how well do typicality measures extracted from a bert text classifier match human judgments of genre typicality? *Sociol. Sci. 2023 March; 10: 82-117* (2023).
2. I Erev, AE Roth, RL Slonim, G Barron, Learning and equilibrium as useful approximations: Accuracy of prediction on randomly selected constant sum games. *Econ. Theory* **33**, 29–51 (2007).
3. R Durand, PA Kremp, Classical deviation: Organizational and individual status as antecedents of conformity. *Acad. Manag. J.* **59**, 65–89 (2016).
4. EG Pontikes, MT Hannan, An ecology of social categories. *Sociol. Sci.* **1**, 311–343 (2014).
5. EB Smith, Identities as lenses: How organizational identity affects audiences' evaluation of organizational performance. *Adm. Sci. Q.* **56**, 61–94 (2011).
6. HP Luhn, A statistical approach to mechanized encoding and searching of literary information. *IBM J. research development* **1**, 309–317 (1957).
7. J Pennington, R Socher, CD Manning, Glove: Global vectors for word representation in *Empirical Methods in Natural Language Processing (EMNLP).* pp. 1532–1543 (2014).
